# Lipidomics changes in bronchoalveolar lavage fluid of refractory mycoplasma pneumoniae pneumonia: LC-MS-based analysis of potential biomarkers and pathogenesis

**DOI:** 10.3389/fped.2025.1655289

**Published:** 2025-09-26

**Authors:** Miao Ma, Jiao Shi, Weigang Wang, Haijuan Huang, Xiaofei Li

**Affiliations:** ^1^ Department of Pediatric Respiratory Medicine, Northwest Women's and Children’s Hospital, Xi'an, Shaanxi, China; ^2^ Statistics and Medical Records Department, Northwest Women's and Children’s Hospital, Xi’an, Shaanxi, China

**Keywords:** children, mycoplasma pneumoniae, bronchoalveolar lavage fluid, lipid metabolism, biomarkers

## Abstract

**Background:**

Previous studies have reported the application of metabolomics in infectious diseases, but little is known about the potential function of bronchoalveolar lavage fluid (BALF) metabolites in children with (mycoplasma pneumoniae pneumonia) MPP and (refractory mycoplasma pneumoniae pneumonia) RMPP.

**Methods:**

In this study, untargeted lipidomic analysis of BALF in MPP patients (*n* = 14) and RMPP patients (*n* = 14) was performed based on the liquid chromatograph-mass spectrometry (LC-MS) method. Orthogonal Projections to Latent Structures Discriminant Analysis (OPLS-DA) was applied to analyze the resultant dataset. Differential metabolites with variable importance in the projection (VIP) >1.5, *P* < 0.05, and |log2FC| > 1 were subjected to multivariate receiver operating characteristic (ROC) analysis to determine the discriminatory power and reliability of potential biomarkers. The potential significance of the differential metabolites was further demonstrated by correlation analysis between the identified differential markers and clinical inflammatory and cardiovascular indicators.

**Results:**

Thirteen differential lipids were found between RMPP and MPP, among which there were three significantly up-regulated differential metabolites (DG(34:4e), PC(36:5), SM(d38:3)), and the areas under the curves (AUCs) of all of them were greater than 0.8, and among the up-regulated differential metabolites of lipids between RMPP and MPP, DG(34:4e) and SM(d38:3) were positively correlated with Hydroxybutyrate Dehydrogenase (HBDH), Lactate Dehydrogenase (LDH), Creatine Kinase (CK), D-Dimer, and Fibrin Degradation Products (FDP).

**Conclusion:**

This is the first study to analyze the lipidomic features of BALF to differentiate between RMPP and MPP. The lipidomics of BALF in patients with RMPP was significantly altered and closely correlated with clinically relevant indicators. These data may contribute to the understanding of the pathogenesis of RMPP and the discovery of new biomarkers and therapeutic targets for RMPP.

## 1 Introduction

Mycoplasma pneumoniae (MP) is a common pathogen in community-acquired pneumonia (CAP) in children (1). About 40% of CAP is caused by MP infection, which ultimately develops into Mycoplasma pneumoniae pneumonia (MPP) (2, 3), which is often manifested as fever, cough, asthma, and respiratory dyspnea. MPP can develop throughout the year, with epidemic peaks occurring every 3–7 years. In 2023, an epidemic peak of MPP was observed in China in the fall and winter seasons, and the number of children with refractory Mycoplasma pneumoniae pneumonia (RMPP) has increased significantly compared with the past. RMPP can present with complications such as pulmonary atelectasis, pulmonary necrosis, bronchiolitis obliterans, and myocardial damage, which brings a heavy economic burden to families and society (4, 5), so early recognition and timely and effective treatment are essential for the prevention of complications. A variety of factors can lead to the progression of MPP to RMPP, including excessive immune response, immune escape, macrolide resistance, and mixed infections with multiple microorganisms (6). Due to the complex pathogenesis of RMPP, the timely diagnosis and treatment of RMPP are limited. There is an urgent need for new diagnostic tools-faster, more reliable, more robust, and less invasive-to differentiate RMPP from common MPP, thus providing a theoretical basis for early diagnosis of RMPP as well as individualized treatment.

Lipid metabolism involves the study of the lipidome or total lipid content of a cell, organ or biological system (7), and aims to identify early biomarkers for disease diagnosis and to understand systemic changes due to complex diseases, which can help to understand and discover the underlying pathogenesis of diseases, and is associated with the development and progression of MP infections (8). Since bronchoalveolar lavage fluid (BALF) is known as “liquid lung biopsy”, lipidomic analysis of BALF is an important strategy to reflect the changes in the lung microenvironment interfered by external factors (9), and it is used to observe lipid phenotypes, to explore the potential biomarkers of RMPP, and to elucidate the biological mechanisms of respiratory diseases.

Therefore, the primary objective of this study is diagnostic, aiming to identify reliable lipidomic biomarkers in BALF that can differentiate between RMPP and common MPP, and provides a more reliable and objective method for early diagnosis of RMPP, potentially improving the prognosis of children with RMPP. While previous studies have applied metabolomics or serum lipidomics to infectious diseases, investigations specifically focusing on BALF lipidomics in MPP and RMPP remain scarce. In particular, no systematic analysis has been conducted to clarify whether alterations in BALF lipid metabolism are associated with the development of refractory disease, nor whether these changes may provide clinically relevant indicators to guide early recognition and individualized treatment. Thus, our study not only provides novel insights into the pathogenesis of RMPP but also attempts to bridge the gap in the current literature by evaluating the diagnostic potential of BALF lipidomic profiling in distinguishing between MPP and RMPP.

## 2 Materials and methods

### 2.1 Objects of study

Patients and the control group were recruited from the Northwest Women's and Children's Hospital from October to December 2023. Inclusion criteria for MPP were as follows: (1) age 1–12 years old; (2) fulfilling the diagnostic criteria for CAP with symptoms of respiratory infection and chest imaging feature suggestive of pneumonia with or without pleural effusion; (3) single serum MP antibody titer ≥1:160 (PA); and a double serum MP antibody titer rises 4-times or more. or positive for MP-DNA or RNA. Children diagnosed with MPP will be diagnosed with RMPP if, after 7 or more days of treatment with macrolide antibiotics, they have a persistently elevated temperature, a persistent worsening of clinical symptoms and chest imaging manifestations, and extrapulmonary complications. Exclusion criteria are as follows: (1) do not meet the inclusion criteria or have incomplete clinical data; (2) patients with congenital heart disease, tuberculosis infection, bronchial foreign body, bronchiectasis, diffuse interstitial lung disease, bronchopulmonary dysplasia, metabolic disease, connective tissue disease, inflammatory bowel disease, congenital immunodeficiency disease, and cystic fibrosis; and (3) use of hormones, or other medications affecting metabolism within 1 month.

Based on the inclusion and exclusion criteria, 28 study subjects were finally included (14 in the MPP group and 14 in the RMPP group). Demographic and clinical characteristics, laboratory and imaging findings collected from all the children enrolled in the study within 24 h of hospital admission, including: chief complaint, duration of hospitalization, fever, cough, wheezing, chest radiograph, Chest computed tomography (chest CT), C-reactive protein (CRP), White blood cell count (WBC), Procalcitonin (PCT), Erythrocyte sedimentation rate (ESR), Neutrophil percentage (NEUT%), Lymphocyte percentage (LYMPH%), HBDH, LDH, CK, Creatine kinase-MB (CK-MB), Aspartate aminotransferase (AST), Alanine aminotransferase (ALT), Gamma-glutamyl transferase (GGT), Albumin (ALB), Total bilirubin (TBIL), Direct bilirubin (DBIL), Indirect bilirubin (IDBIL), Alkaline phosphatase (ALP), Total protein (TP), Platelet count (PLT), Hemoglobin (HGB), D-Dimer, and FDP.

To minimize confounding factors, all included RMPP patients met the diagnostic criteria for primary Mycoplasma pneumoniae pneumonia. Patients with documented secondary bacterial or viral infections were excluded based on microbiological testing and clinical evaluation, consistent with the Chinese guidelines. During hospitalization, all enrolled patients received standard supportive care. Oxygen therapy was limited to low-flow nasal cannula or high-flow nasal oxygen (HFNO) as clinically indicated. None of the patients required non-invasive ventilation (NIV) or invasive mechanical ventilation at the time of BALF collection. This ensured that the observed lipidomic changes were not confounded by ventilation-related factors such as oxygen toxicity or ventilator-associated pneumonia.

The study followed the Declaration of Helsinki and was approved by the Northwest Women's and Children's Medical Ethics Committee, Ethics No. 2024–022.

### 2.2 BALF sample collection and pre-processing

CT scan and bronchoscopy were performed within 72 h of admission. The necessity and feasibility of bronchoscopy were determined on a case-by-case basis by an experienced clinician. According to the results of the CT scan, sterile saline was injected into the lesion sites of children with MPP and RMPP, and if the lesions were extensive, the lingual lobe of the left lung or the middle lobe of the right lung was selected for irrigation. Specific operations were performed according to the Chinese Children's Bronchoscopy Guidelines (2018 edition) for bronchoscopy and BALF collection. The collected BALF was clarified by centrifugation at 10,000 × g for 10 min, and the supernatant and sediment were separated and frozen (−80 °C) until the time of testing. In accordance with the Chinese Children's Bronchoscopy Guidelines (2018 edition), the volume of sterile saline instilled for bronchoalveolar lavage was determined according to the child's body weight, at approximately 1 ml/kg per aliquot, with a total lavage volume not exceeding 3–5 ml/kg. For most children enrolled in this study, the actual instilled volume ranged between 10 and 20 ml, depending on age and weight. The lavage was performed in aliquots to reduce the risk of procedure-related complications. Recovered BALF volumes were recorded, and the supernatant after centrifugation was used for lipidomic analysis. This approach minimizes variability caused by dilution and ensures comparability of lipid concentrations across patients.

### 2.3 Untargeted lipidomic analysis based on LC-MS

Lipid extraction (10, 11): the volume needed to reach a protein amount of 250 μg for DTT-treated BALF into a 2 ml centrifuge tube, add 750 μl chloroform methanol solution (2:1, v/v), whirl and shake for 30 s; place on ice for 40 min, add 190 μl H_2_O, mix and shake for 30 s, and let place on ice for 10 min; Centrifuge at 12,000 rpm for 5 min at room temperature, take 300 μl of organic layer, transfer to a new 2 Add 500 μl of chloroform mixture (2:1, v/v), whirl and shake for 30 s; Centrifuge the sample at 12,000 rpm for 5 min, take 400 μl of the organic layer and transfer it to a new 2 ml centrifuge tube, and then concentrate the sample with a vacuum concentrator; dissolve the sample with 200 μl of isopropanol and filter it through a 0.22 μm membrane to obtain the sample to be measured and subjected to LC-MS. Machine detection (12, 13): 1. Chromatographic conditions: ACQUITY UPLC® BEH C18 1.7 µm (2.1 × 100 mm), with the autosampler temperature set to 8°C, and 2 μl of sample was injected for gradient elution at a flow rate of 0.25 ml/min and a column temperature of 50°C. The mobile phase was: acetonitrile: water = 60:40 (0.1% formic acid +10 mm ammonium formate) (A2)—isopropanol: acetonitrile = 90:10 (0.1% formic acid +10 mm ammonium formate) (B2). The gradient elution program was 0–5 min, 70%–57% A; 2; 5–5.1 min, 57%–50% A2; 5.1–14 min, 50%–30% A2; 14–14.1 min, 30% A2; 14.1–21 min, 30%–1% A2; 21–24 min, 1% A2; 24–24.1 min, 1%–70% A2; 24.1–28 min, 70% A2. Spectrometry conditions: the instrument: electrospray ionization (ESI), positive and negative ion ionization modes, with a positive ion spray voltage of 3.50 kV, a negative ion spray voltage of 2.50 kV, a sheath gas of 30 arb, and an auxiliary gas of 10 arb. The capillary temperature was set at 325 ℃, and the full scan was carried out with a resolution of 35,000, with a scanning range of 150–2,000, and the HCD was used to measure the mass concentration in the range of 150–2,000 ℃. The capillary temperature was 325 ℃, the full scan was performed at a resolution of 35,000 with a scanning range of 150 ∼2,000, and HCD was used for secondary cleavage with a collision voltage of 30 eV, while dynamic exclusion was used to remove unnecessary MS/MS information.

## 3 Statistical analysis

### 3.1 Statistical analysis of clinical data

Clinical data were analyzed by JASP 0.19, and data were expressed as mean ± standard deviation. The two groups of data were subjected to the “normality test”, and continuous data were analyzed by Student's *t*-test if they met the normality, and rank-sum test if they did not meet the normality. Categorical data were analyzed by the chi-square test. The significance level was set at *P* < 0.05.

### 3.2 Statistical analysis of lipidomics

Total peak area normalization based on the signal response within the sample, enabling inter-batch data correction to eliminate instrument batch errors. Substances with RSD > 30% in QC samples are filtered out.

The sample data were analyzed by principal component analysis (PCA), partial least squares discriminant analysis (PLS-DA), and Orthogonal Projections to Latent Structures Discriminant Analysis (OPLS-DA) of the R software package Ropls respectively. And score plots, loading plots and S-plot plots were plotted to demonstrate the differences in lipid composition among samples, respectively. The permutation test was performed on the model for the fitting test. R2X and R2Y denote the explanatory rate of the constructed model to the X and Y matrices, respectively, and Q2 labels the predictive ability of the model, and the closer their values are to 1 indicates that the better the model fits and the more accurately samples in the training set can be classified into their original attributions. Based on the statistical test to calculate the *P* value, the OPLS-DA dimensionality reduction method to calculate the VIP, and fold change to calculate the multiple of differences of the components, we measured the influence intensity and the interpretation ability of the content of each lipid component on the classification discrimination of the samples, and assisted in marking the screening of lipids. Lipid molecules were considered statistically significant when the *P* value < 0.05 and VIP value > 1.

Lipids were analyzed for carbon chain length and carbon saturation by the R lipidomoR software package. Differential lipids were also analyzed for functional enrichment by the R LION software package. Lipid functional enrichment analyses were performed by the LION lipid ontology database (14). The LION database associates more than50,000 lipids with four major branches including: (1) lipid classification (LIPIDMAPS classification hierarchy); (2) chemical and physical properties (fatty acid length and unsaturation, functional group charge, membrane fluidity, etc.); (3) function; and (4) subcellular components (major subcellular localization).

To ensure comparability, an equal volume of BALF (200 μl) was extracted for each sample, thereby minimizing technical variation in total lipid load. All peak areas were normalized to the total ion current within each sample to obtain relative percentages. While absolute lipid quantification was not performed in this study, the consistent input volume across samples provides confidence that the observed differences primarily reflect biologically relevant alterations rather than technical bias. In future work, incorporation of internal standards and absolute quantification will further improve the robustness of lipid concentration comparisons.

For lipid identification, we applied a two-step approach: (1) preliminary annotation was based on accurate mass measurements against public lipid databases (e.g., LIPIDMAPS); (2) putative identifications were further confirmed using MS/MS fragmentation patterns, including characteristic product ions and neutral loss information, to ensure structural reliability of the assignments. Only metabolites with consistent MS/MS spectra were retained for downstream analysis.

### 3.3 Selection of biomarkers

We further refined the screening criteria to screen for differentially expressed lipid metabolites, with stringent screening criteria of VIP > 1.5 and *P*-value < 0.05, |log_2_FC| > 1. We then assessed and validated the accuracy of the differentially expressed metabolites by ROC curves and AUC. Finally, the correlation analysis between clinical indicators and differential metabolites was performed and a heat map was plotted.

## 4 Results

### 4.1 Demographic and clinical characteristics of participants

The demographic characteristics of the patients are shown in Table 1. There were no significant differences in age as well as gender between the two groups of children in Table 1. The laboratory parameters of the 28 enrolled children are shown in Table 2. The correlation of the blood tests of the two groups of children in Table 2 showed that PCT, NEUT%, HBDH, LDH, CK, AST, ALT, D-Dimer, and FDP were significantly higher in the RMPP group than in the MPP group (*P* < 0.05). In contrast, LYMPH%, ALB, ALP, and TP were significantly lower in the RMPP group than in the MPP group (*P* < 0.05).

**Table 1:** Demographic characteristics of enrolled children.

Characteristics	MPP (*n* = 14)	RMPP (*n* = 14)	*P*-value^a^
Gender (male/female)	7/7	9/5	0.445
Age (years)	±5.98 2.58	±6.28 1.38	0.711
Age range (years)	1−7	3–12	/

^a^ *P* < 0.05.

MPP, mycoplasma pneumoniae pneumonia; RMPP, refractory mycoplasma pneumoniae pneumonia.

**Table 2:** Laboratory results comparison of MPP and RMPP.

Variables	RMPP (*n* = 14)	MPP (*n* = 14)	T (Z)	*P*-value^a^
CRP	22.14 (8.49,55.1)	10.54 (6.42,20.81)	−1.542	0.123
WBC	7.10 ± 3.11	6.59 ± 2.92	0.450	0.657
PCT	0.5 (0.16,1.2)	0.14 (0.07,0.27)	−2.851	0.004
ESR	38 (27.25,57.25)	26 (15.75,37.5)	−1.839	0.066
NEUT%	72.44 ± 9.08	59.09 ± 14.12	2.974	0.006
LYMPH%	21.03 ± 9.97	32.71 ± 12.73	−2.70	0.012
HBDH	417.97 ± 81.76	192.42 ± 33.74	9.541	0.000
LDH	574.56 ± 104.4	257.5 ± 46.14	10.393	0.000
CK	191.98 (73.93,491.21)	64.86 (52.13,89.95)	−2.389	0.017
CK-MB	19.34 ± 6.98	17.39 ± 6.5	0.766	0.453
AST	59.06 (39.56,95.02)	26.29 (20.67,30.04)	−3.722	0.000
ALT	28.91 (19.83,58.83)	11.71 (9.97,14.62)	−3.492	0.000
GGT	13.19 ± 4.13	11.63 ± 1.94	1.279	0.212
ALB	34.64 ± 4.49	41.48 ± 2.88	−4.799	0.000
TBIL	7.54 ± 1.63	6.85 ± 2.11	0.975	0.339
DBIL	1.51 (1.16,1.63)	1.33 (1.05,1.41)	−1.886	0.059
IDBIL	6.04 (4.76,6.95)	5.73 (3.8,6.05)	−1.103	0.270
ALP	113.48 ± 24.66	164.2 ± 33.53	−4.559	0.000
TP	61.49 ± 6.03	69.38 ± 3.37	−4.275	0.000
PLT	254.79 ± 77.7	301.21 ± 119.9	−1.216	0.235
Hgb	124.71 ± 5.88	128.07 ± 8.08	−1.257	0.220
D-dimer	2.54 ± 0.82	0.5 ± 0.2	9.034	0.000
FDP	8.33 ± 3.33	2.35 ± 0.89	6.492	0.000

^a^ *P* < 0.05.

MPP, mycoplasma pneumoniae pneumonia; RMPP, refractory mycoplasma pneumoniae pneumonia; CRP, C-reactive protein; WBC, white blood cell count; PCT, procalcitonin; ESR, erythrocyte sedimentation rate; NEUT%, neutrophil percentage; LYMPH%, lymphocyte percentage; HBDH, hydroxybutyrate dehydrogenase; LDH, lactate dehydrogenase; CK, creatine kinase; CK-MB, creatine kinase-MB; AST, aspartate aminotransferase; ALT, alanine aminotransferase; GGT, gamma-glutamyl transferase; ALB, albumin; TBIL, total bilirubin; DBIL, direct bilirubin; IDBIL, indirect bilirubin; ALP, alkaline phosphatase; TP, total protein; PLT, platelet count; Hgb, hemoglobin; FDP, fibrin degradation products.

### 4.2 General description of lipidomics of BALF samples

Untargeted lipidomic analysis of 28 BALF samples was performed by LC-MS to generate BALF lipid profiles of 28 children, and 480 lipid compounds were detected in these specimens, which were categorized according to the lipid chain and moiety as BisMePA, Cer, ChE, CL, Co, TG, DG, dMePE, GM2, GM3, Hex1Cer, Hex2Cer, LdMePE, PC, PE, MePC, MGDG, PG, PI, PS, SM, SPH, StE and other categories (Supplementary Figure S1, Supplementary Table S1). In the BALF samples, the percentage of the top 10 lipids included PC contributing to the total lipid signaling with 26.42%, followed by TG (15.09%), PE(13.21%), MePC(12.37%), SM(10.48%), Cer(7.55%), Hex2Cer(4.61%), BisMePA(3.98%), PG(3.35%), PI(2.94%).

### 4.3 Biomarker selection for RMPP

**Figure 1:**
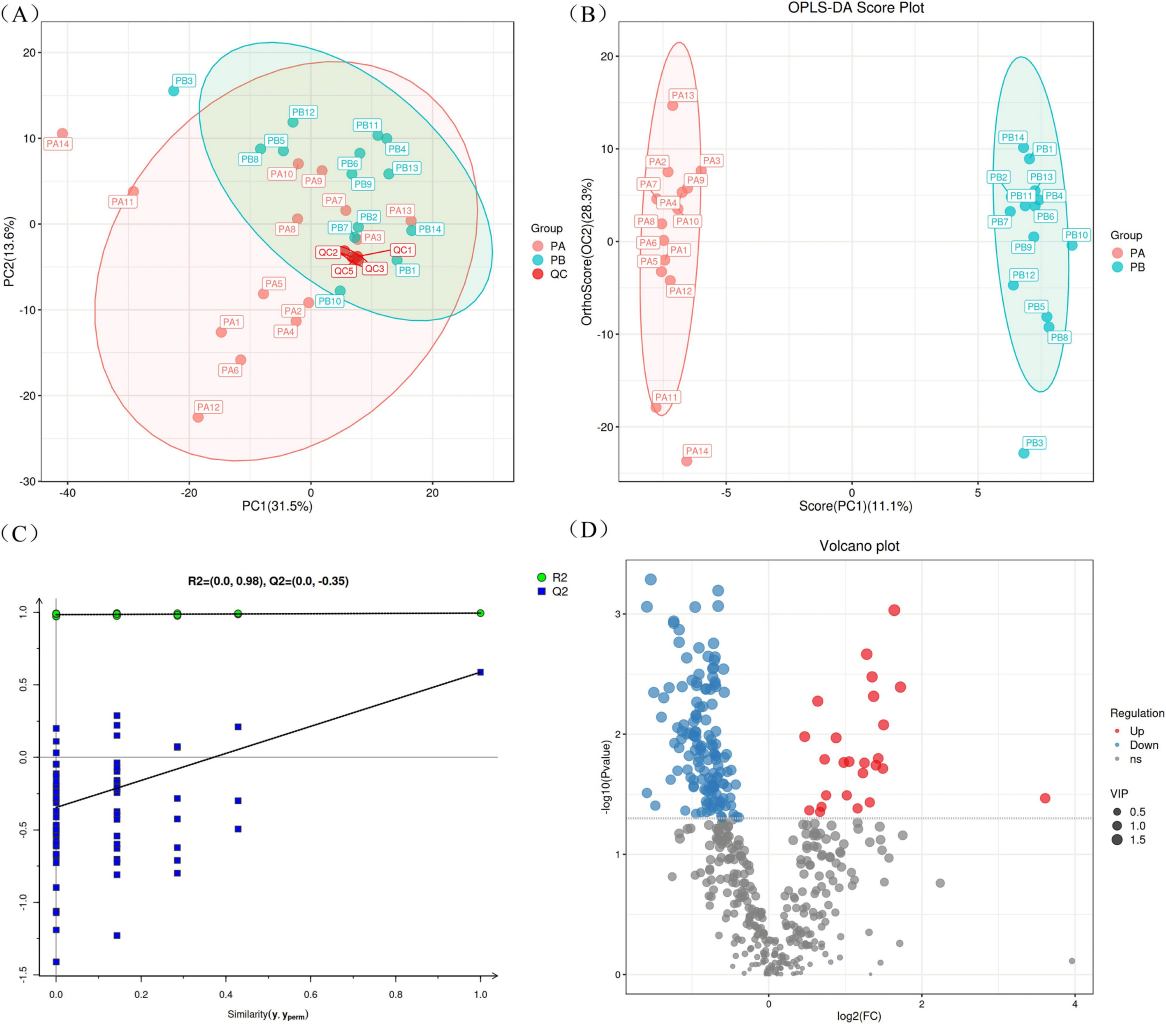
LC/MS analysis of BALF specimens. **(A)** Represents the PCA score plot for quality control. **(B)** Represents the OPLS-DA score plot of BALF samples from children with RMPP and MPP. **(C)** Represents the OPLS-DA model permutation test plot in BALF specimens. **(D)** Displays a volcano diagram showing the differences in lipid compounds between RMPP and MPP children. BALF, bronchoalveolar lavage fluid; RMPP (PA), refractory mycoplasma pneumoniae pneumonia; MPP (PB), mycoplasma pneumoniae pneumonia.

To ensure the reliability of the results, a PCA score plot was generated (Figure 1A), which included the PA group (RMPP), the PB group (MPP), and the QC samples. The QC samples (in red color) were tightly clustered, which indicated high instrumental stability and good quality of LC-MS-based data. In addition, the QC samples have a tendency to cluster within the 95% confidence interval, again indicating the reliability of the analytical method.

**Figure 2:**
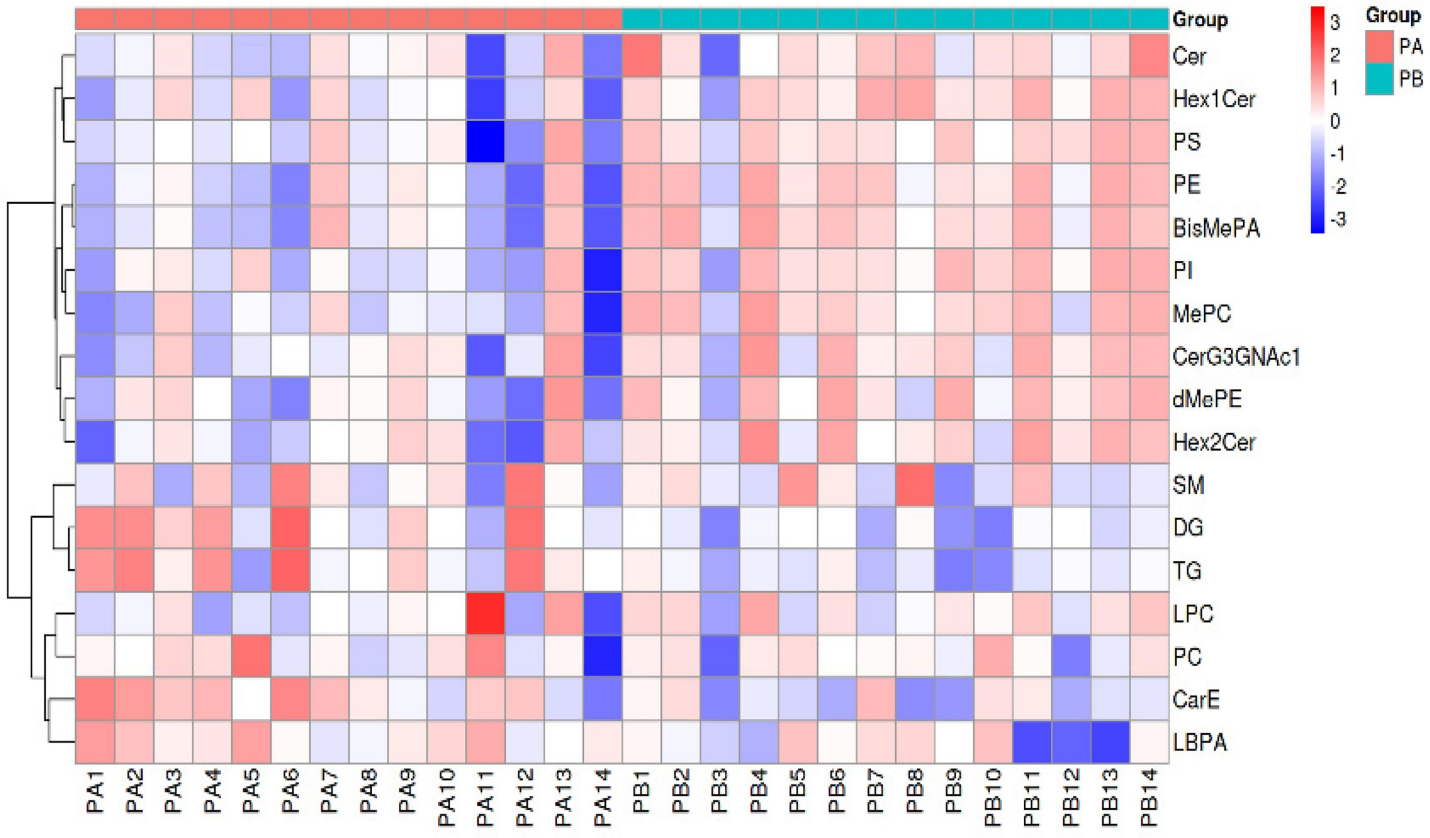
Metabolite identification heatmap. The heat map displays the relative abundance of identified lipid compounds in each BALF sample. BALF, bronchoalveolar lavage fluid; RMPP(PA), refractory mycoplasma pneumoniae pneumonia; MPP(PB), mycoplasma pneumoniae pneumonia.

In order to better display the subtle differences between the two groups of samples, multivariate statistical methods such as PCA, PLS-DA and OPLS-DA analysis were used based on the analysis method of LC-MS. Among them, OPLS-DA analysis maximally demonstrated the metabolite differences between the two groups of samples, and the score plot showed that there was a clear separation between RMPP patients and MPP patients (Figure 1B). Subsequently, the permutation test of the OPLS-DA model further showed good fitting and predictive power (Figure 1C). The volcano plot showed differences in metabolite screening in children with RMPP relative to those with MPP (Figure 1D). Lipid molecules were considered statistically significant when the VIP > 1, *P* < 0.05, and of the 480 lipid compounds analyzed, 168 lipid compounds met these criteria (Supplementary Table S2). Of these, 25 lipid compounds were up-regulated in the RMPP group, and 143 lipid compounds were down-regulated in the RMPP group. The heat map further shows the meaningful distribution of lipids identified in each sample (Figure 2).

To further determine which differentially expressed lipid compounds played the greatest role in distinguishing between RMPP and MPP, we refined the screening criteria by selecting differential metabolites when VIP > 1.5, *P*-value < 0.05, and |log_2_FC| > 1. Based on these criteria, we found a total of 17 lipid compounds with different abundance in the RMPP group compared to the MPP group (Table 3).

**Table 3:** Identification and selection of differentially expressed lipid compounds.

Accession	Class	log2FC	*P*. value	VIP	AUC	rtmin	IonFormula	Regulation
BisMePA (18:2e_18:2)	BisMePA	−1.24	0.001149	1.726002	0.83	14.8849	c41 h79 o7 n1 p1	Down
BisMePA (20:2e_20:4)	BisMePA	−1.17	0.001351	1.709927	0.84	16.06433	c45 h83 o7 n1 p1	Down
DG (18:2_18:2)	DG	−1.3	0.004111	1.626393	0.80	13.856	C39 H72 O5 N1	Down
DG (34:4e)	DG	1.28	0.00216	1.675035	0.82	20.57262	C37 H67 O4	Up
DG (36:4e)	DG	1.35	0.003336	1.594066	0.76	20.91962	C39 H71 O4	Up
Hex2Cer (d18:1_22:0)	Hex2Cer	−1.03	0.00976	1.509652	0.81	16.47901	c52 h100 o13 n1	Down
MePC (40:6e)	MePC	−1.59	0.000871	1.779762	0.84	17.07473	C49 H88 O7 N1 P1 Na1	Down
PC (30:1)	PC	−1.37	0.004981	1.553367	0.78	11.352	c38 h75 o8 n1 p1	Down
PC (36:5)	PC	1.37	0.004843	1.626269	0.85	12.49152	c44 h79 o8 n1 p1	Up
PE (18:0p_18:2)	PE	−1.24	0.001191	1.72153	0.83	14.9177	c41 h79 o7 n1 p1	Down
PE (20:0p_20:4)	PE	−1.17	0.001719	1.676276	0.84	16.042	c45 h83 o7 n1 p1	Down
PE (20:2e_20:4)	PE	−1.13	0.004024	1.565062	0.82	14.73773	c45 h79 o7 n1 p1	Down
PE (42:6e)	PE	−1.54	0.000516	1.859008	0.84	15.943	C47 H84 O7 N1 P1 Na1	Down
SM (d38:3)	SM	1.64	0.000929	1.802386	0.83	14.494	c43 h84 o6 n2 p1	Up
SM (d43:6)	SM	−1.5	0.0045	1.599087	0.81	12.717	c48 h88 o6 n2 p1	Down
tg (16:0_18:1_18:2)	TG	1.72	0.004057	1.588917	0.80	20.56133	C55 H104 O6 N1	Up
dMePE (40:6e)	dMePE	−1.07	0.002319	1.635085	0.83	15.93319	c47 h83 o7 n1 p1	Down

VIP, variable importance in projection; AUC, area under curve; DG, diacylglycerol (DG); PE, phosphatidylethanolamine; PC, phosphatidylcholine; PA, phosphatidic acid; SM, sphingomyelin; Cer, ceramides.

In addition, we plotted the ROC for the lipid compounds screened by VIP, |log2FC|, and *P* values. The closer the apex of the curve is to the upper left corner, the stronger the discriminatory ability is and the higher the AUC is. The AUC value is used to measure the difference in differential compounds between RMPP and MPP with good sensitivity and specificity, which is clinically important in the diagnosis of diseases. Among these differential compounds, a total of 13 lipid compounds with AUC > 0.8 were screened (Table 3, Supplementary Figure S2), of which DG(34:4e), PC(36:5), and SM(d38:3) were up-regulated, and BisMePA(18:2e_18:2), BisMePA(20:2e_20:4), Hex2Cer(d18:1_22:0), MePC(40:6e), PE(18:0p_18:2), PE (20:0p_20:4), PE(20:2e_20:4), PE(42:6e), SM(d43:6), and dMePE (40:6e) down-graded.

### 4.4 Lipid structure analysis shows the distribution pattern of its components

**Figure 3:**
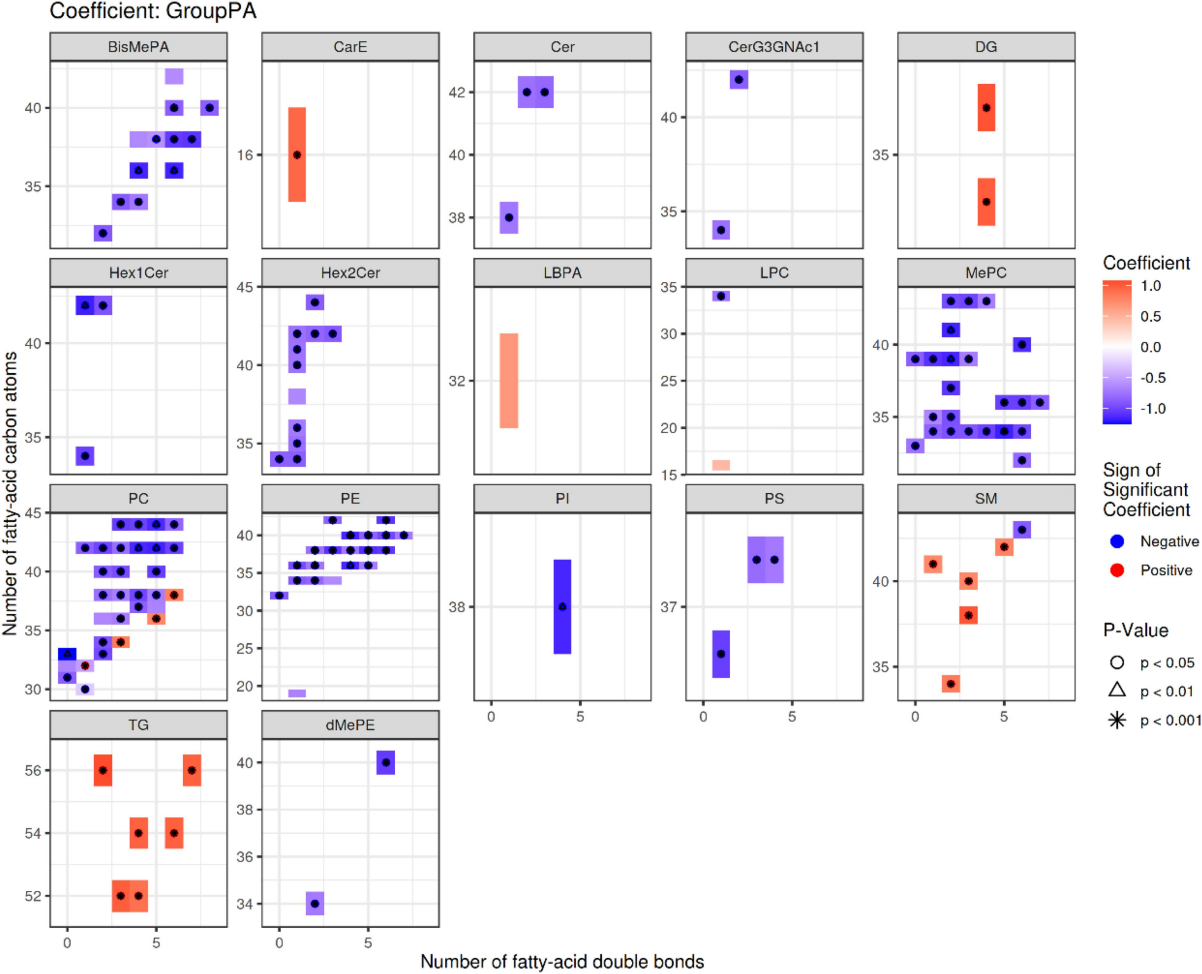
Statistical heatmap of differences in lipid structure characteristics. Each small graph represents the differences in lipid structure characteristics of a single group under a lipid classification. The *X*-axis represents the level of carbon saturation (number of double bonds), and the *Y*-axis represents the number of lipid carbon atoms. In the heat map, rectangles represent different lipids of the same classification, colors indicate significant differences in expression, red indicates significant up-regulation (high differential lipid levels in the RMPP group), and blue indicates significant down-regulation (low differential lipid levels in the RMPP group). Lipids with significant statistical differences between the comparison groups are highlighted with different symbols.

**Figure 4:**
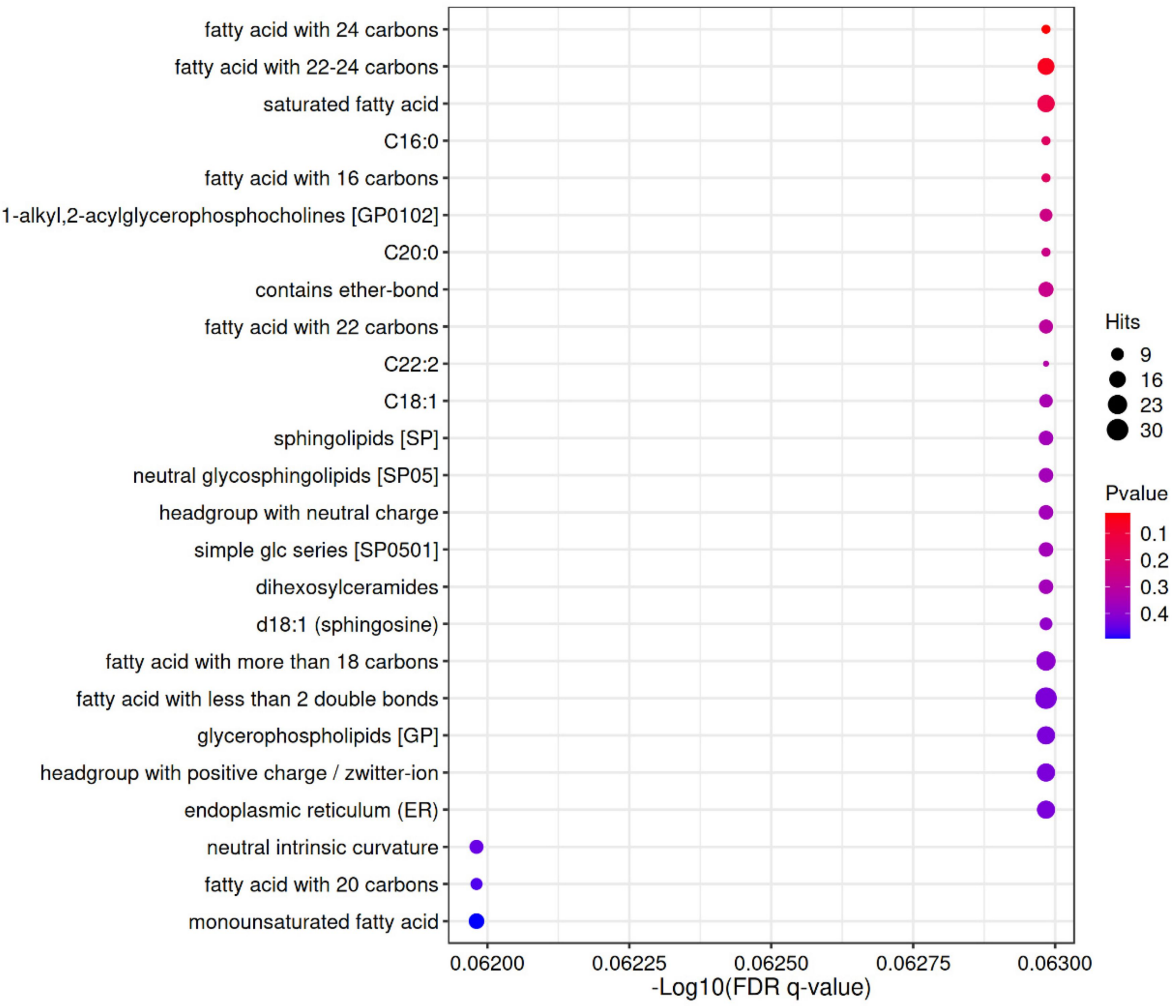
LION enrichment bubble. The horizontal axis represents the—log10 value of enriched FDR q-value; The vertical axis represents the names of structures, functions, etc. in the enriched LION database; The color indicates the significance level of *P* value enrichment, and the redder the color, the more significant the enrichment result; The size of the bubble indicates the number of differential lipids enriched to that entry, with a larger size indicating more enriched lipids.

In the following sections, we first present the descriptive results of lipid structure characteristics and enrichment analysis, and then indicate which observations are based on direct quantitative lipidomic data and which represent functional inferences derived from enrichment analysis. Specifically, quantitative data such as fold changes and relative abundances are derived directly from LC-MS measurements, whereas terms such as “membrane fluidity” or “signaling” represent functional implications inferred from enrichment results and previous literature (15). We next analyzed the structural features of the differential lipid results obtained from the identification (Figure 3). The up-regulated lipids were dominated by DG and PC, with PC(36:5) having a high number of double bonds (5 unsaturated bonds), suggesting that it may influence disease progression by increasing membrane fluidity or participating in inflammatory signaling (e.g., arachidonic acid metabolism) (16). The significant elevation of DG(34:4e) may be associated with enhanced biosynthesis of long-chain fatty acids (22–24 carbon). This association was further verified by LION enrichment analysis (Figure 4). Down-regulated lipids showed a decrease in ether-bonded BisMePA (e.g., BisMePA(18:2e_18:2)) and long-chain Hex2Cer(d18:1_22:0), possibly reflecting impaired alveolar membrane stability or disturbed sphingolipid metabolism.

### 4.5 Enrichment analysis of different lipid metabolites in RMPP, MPP

Lipid metabolism-related pathways were significantly enriched overall (up-regulated in red, down-regulated in purple), with a total of 20 lipid categories significantly enriched (*P*-value < 0.05), with lipids with fatty acid chain lengths > 22 being the most significantly enriched (17 entries). The enrichment of the up-regulated lipid DG(34:4e) was closely associated with the biosynthesis of saturated fatty acids (18 entries) and 22–24 carbon fatty acids (17 entries). Down-regulated lipids: deletion of SM(d38:3) corresponds to a decrease in Sphingolipids [SP] (12 deletions) and C22:2 (4 deletions). down-regulation of BisMePA(18:2e_18:2) is consistent with headgroup with neutral charge (12 deletions). Chain length and saturation: enrichment of long-chain fatty acids (e.g., 22–24 carbon) and monounsaturated fatty acids (monounsaturated fatty acid, 14 enrichment) may suggest altered membrane fluidity. Specific lipid categories: dysregulation of sphingolipids (SP, 12 deletions) and ether-bonded lipids (13 enrichment) may be associated with abnormal cell signaling. Up-regulated lipids were mainly associated with long-chain fatty acids (e.g., 22–24 carbons) and monounsaturated fatty acids (-Log10(FDR-q) = 5), whereas down-regulated lipids were concentrated in sphingolipids (SP, -Log10(FDR-q) = 12) and ether-bonded lipids. It is important to note that “functional enrichment” here refers to the statistical over-representation of certain lipid categories or structural features (e.g., long-chain fatty acids, ether lipids) among the differential metabolites, and does not imply a direct increase or decrease in absolute lipid abundance. For clarity, in the enrichment output, the term “entries” denotes the number of differential lipids mapping to a given functional category in the LION database, whereas “deletions” indicates the number of expected entries that were absent or down-regulated compared with the reference background. These clarifications help to avoid misinterpretation and distinguish statistical enrichment from direct biochemical quantification.

### 4.6 Correlation analysis between differential lipids and clinical laboratory indicators

**Figure 5:**
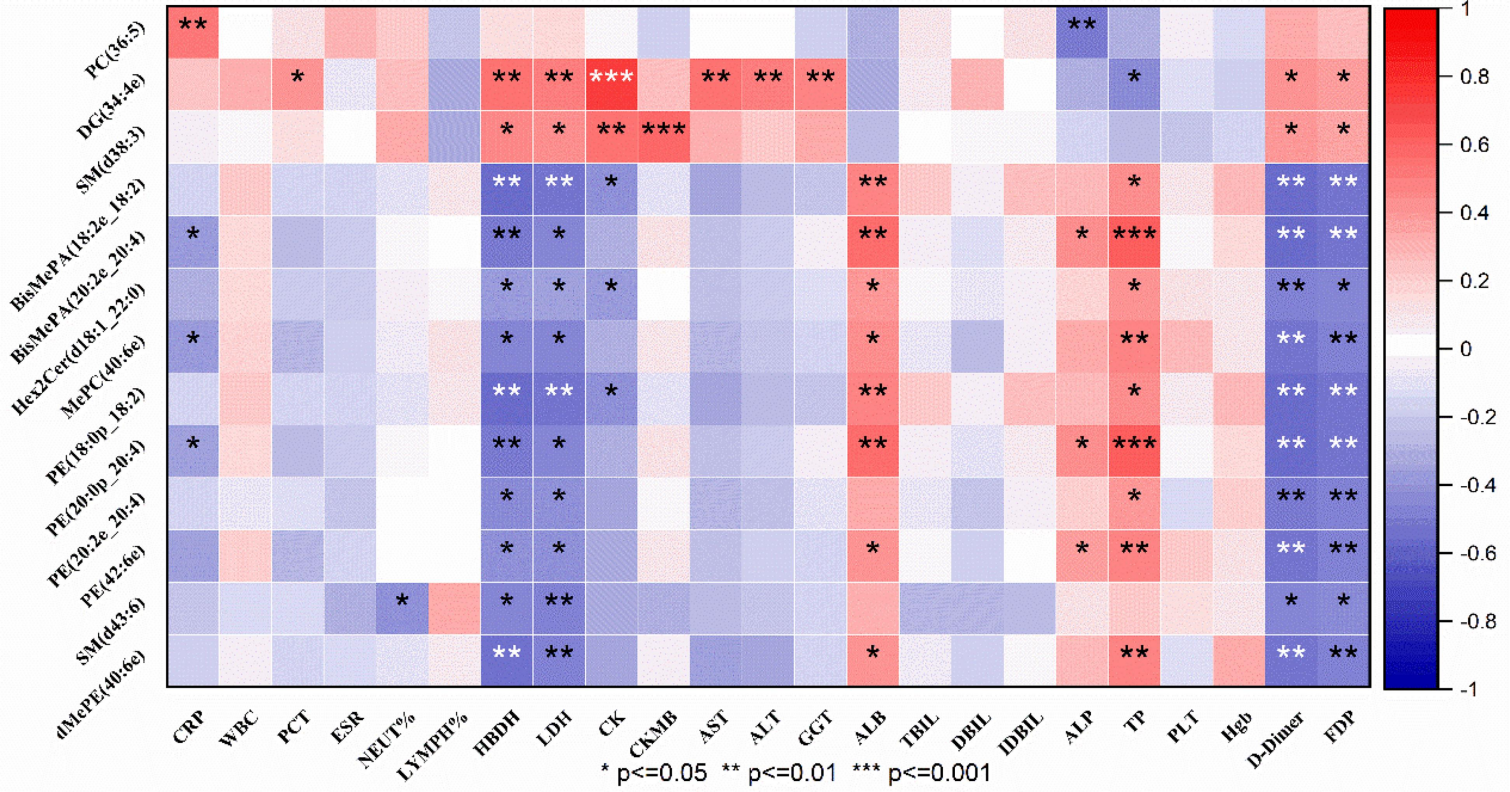
The relationship between differentially expressed metabolites of RMPP and MPP and clinical indicators. Red represents positive correlation, blue represents negative correlation.

Correlation analysis between differential lipids and clinical laboratory indicators was performed using the Spearman rank correlation test. Both correlation coefficients (r) and *P* values were calculated, and only statistically significant associations (|*r*| > 0.4, *P* < 0.05) are highlighted in the heatmap (Figure 5) for clarity. The 13 differential metabolites have shown good potential as biomarkers for diagnosing the condition in children with RMPP. Further studies are needed to determine the potential relationship between the 13 differential metabolites and clinical features, and we investigated the 13 differential metabolites presented in Table 3 in relation to the hematological parameters of the children (CRP, WBC, PCT, ESR, NEUT%, LYMPH%, HBDH, LDH, CK, CK-MB, AST, ALT, GGT, ALB, TBIL, DBIL, IDBIL, ALP, TP, PLT, Hgb, D-Dimer, FDP) (Figure 5). The results showed that many differentially expressed lipid compounds exhibited significant relationships with clinically relevant indicators.

The up-regulated lipid PC (36:5) was positively correlated with CRP and negatively correlated with ALP. The up-regulated lipid DG (34:4e) was positively correlated with PCT, HBDH, LDH, CK, AST, ALT, GGT, D-Dimer, and FDP, while it was negatively correlated with TP. Up-regulated lipid SM (d38:3) was positively correlated with HBDH, LDH, CK, CKMB, D-Dimer, and FDP.

The down-regulated lipid BisMePA (18:2e_18:2) was negatively correlated with HBDH, LDH, CK, D-Dimer, and FDP, while it was positively correlated with ALB and TP. The down-regulated lipid BisMePA (20:2e_20:4) was negatively correlated with CRP, HBDH, LDH, D-Dimer, and FDP, whereas it was positively correlated with ALB, ALP, and TP. The down-regulated lipid Hex2Cer(d18:1_22:0) was negatively correlated with HBDH, LDH, CK, D-Dimer, and FDP, and positively correlated with ALB and TP. The down-regulated lipid MePC(40:6e) was negatively correlated with CRP, HBDH, LDH, D-Dimer, and FDP, whereas it was positively correlated with ALB and TP. Down-regulated lipid PE (18:0p_18:2) was negatively correlated with HBDH, LDH, CK, D-Dimer, and FDP, whereas it was positively correlated with ALB and TP. Down-regulated lipid PE (20:0p_20:4) was negatively correlated with CRP, HBDH, LDH, D-Dimer, and FDP, whereas it was positively correlated with ALB, ALP, and TP. Down-regulated lipid PE (20:2e_20:4) was negatively correlated with HBDH, LDH, D-Dimer, and FDP, while it was positively correlated with TP. The down-regulated lipid PE(42:6e) was negatively correlated with HBDH, LDH, D-Dimer, and FDP, while positively correlated with ALB, ALP, and TP. The down-regulated lipid SM (d43:6) was negatively correlated with NEUT%, HBDH, LDH, D-Dimer, and FDP. The down-regulated lipid dMePE (40:6e) was negatively correlated with HBDH, LDH, D-Dimer, and FDP, and positively correlated with ALB and TP.

The correlation heat map suggested a significant correlation between different lipid categories and clinical hematologic indicators, which, in combination with the previously described significant differences in inflammatory markers in children with RMPP and MPP, suggests that changes in the BALF lipidome in children with RMPP may be related to systemic inflammatory responses.

## 5 Discussion

In recent years, the incidence of RMPP has been increasing and its treatment is difficult. Current studies generally agree that excessive inflammatory response plays a key role in the pathogenesis of RMPP, and early prediction of RMPP has become a focus of research (17). Unlike genomics, transcriptomics and proteomics, only metabolomics can reveal the final downstream products of the inflammatory process. Lipids in metabolomics constitute about 90% of pulmonary surfactant (PS), which is essential for maintaining the small airways and surface tension of alveoli (18). Altered lipid composition in PS is a common feature of several acute and chronic respiratory diseases, and inflammation due to infection triggers marked changes in lipid metabolism of the organism (19); therefore, studying the lipid metabolism of endobronchial secretions or bronchoalveolar lavage fluid changes is an effective means to help explore the pathogenesis and therapeutic targets of respiratory diseases (20). Meanwhile, changes in the metabolome reflect changes in the biochemistry of the host cell following MP infection. MP can take up certain lipids from the surrounding area into their membranes, thereby affecting lipid metabolism. To the best of our knowledge, only a few studies have evaluated the relationship between lipid metabolism and MPP, and in these studies, much attention has been paid to general MPP and serum lipidomics (21–23). This is the first time that the relationship between lipids and RMPP in BALF has been evaluated, as well as the potential associated roles.

In children, lung function is not yet well developed, so age may be an important factor influencing the abundance of lipid compounds (24). Therefore, we performed a statistical analysis of age in the RMPP group and MPP group, and there was no significant difference in age between the two groups, aiming to minimize the effect of age on the abundance of lipid compounds. We also statistically analyzed the gender between the two groups of children, and there was also no significant difference in gender between the two groups.

A total of 13 differential lipids were identified in the BALF of children with RMPP and MPP in this study, focusing on glycerides (diacylglycerol (DG)), glycerophospholipids (phosphatidylethanolamine (PE), phosphatidylcholine (PC), phosphatidic acid (PA)), and sphingolipids (sphingomyelin (SM), ceramides (Cer)). PC, as the most important constituent of the PS, with phospholipase acting on PC, the highly active lysophosphatidylcholine(LPC) can be generated, which has a wide range of pro-inflammatory activities, including the promotion of reactive oxygen cluster production, secretion of chemokines and cytokines, and up-regulation of adhesion molecules (25). Yoder M et al. (26) used LC-MS to comparatively analyze BALF samples from seven asthmatic patients and eight healthy volunteers, and found that LPC (16:0) and LPC (18:0) were significantly elevated in asthmatic patients with moderate lung function impairment, accompanied by increased phospholipase A2 activity. The up-regulation of PC (36:5) and its positive correlation with CRP in the present study further supports its key role in the inflammatory cascade response in RMPP, suggesting that dysregulated lipid metabolism may be involved in the pathomechanism of RMPP disease progression. Pulmonary surfactant is composed of approximately 90% lipids, mainly phosphatidylcholine (PC) and phosphatidylglycerol, which are essential for reducing alveolar surface tension and maintaining lung compliance (27, 28). Alterations in surfactant lipid composition have been reported in multiple inflammatory lung diseases, including asthma and acute respiratory distress syndrome, and are known to influence host defense and inflammatory signaling (29, 30). In this context, our finding of up-regulated PC(36:5) and its positive correlation with CRP is consistent with previous observations that phospholipid dysregulation may exacerbate airway inflammation.

Studies on SM have shown that inflammation induces SM hydrolysis, which tends to reduce the amount of SM, leading to ceramide accumulation (31), nevertheless, enhanced *de novo* sphingolipid biosynthesis during inflammation can lead to ceramide formation and subsequent sphingomyelin formation (32). The inflammatory manifestations of the children with RMPP in the study and the up-regulation of their SM(d38:3) suggest that SM may be involved in the inflammatory pathogenesis of RMPP. In addition, although the sphingolipid signaling pathway plays an important role in the regulation of macrophage growth and survival pathways (33), the exact role of SM in RMPP needs to be further investigated. In addition, ceramides identified in our analysis warrant attention, as they are bioactive sphingolipids that regulate apoptosis, inflammation, and barrier function in the lung. Accumulation of ceramides has been associated with epithelial injury and impaired repair mechanisms in pulmonary diseases (34, 35). Although their exact role in RMPP remains to be elucidated, the observed alterations in ceramide-related metabolites in our study suggest that sphingolipid metabolism may contribute to disease progression and deserve further investigation.

Cer in differential lipids belongs to the class of ceramides, which are the main structural components of sphingolipids and play a crucial role in activating cell signaling after pathogen infection (36). Many sphingolipid types and their components, such as ceramides, play important roles in the regulation of cell signaling such as growth, inflammation, and death, and in conjunction with the alterations in SM described previously, it is therefore not surprising that altered sphingolipid metabolism may be associated with MP pathogenicity (37, 38).

Altered PE levels or metabolic abnormalities are associated with mitochondrial dysfunction, which may trigger or exacerbate inflammation through mechanisms such as reactive oxygen species generation (ROS), inflammatory vesicle activation, or impaired oxidative phosphorylation (OXPHOS), and reductions in BisMePA and PE may have weakened the antioxidant capacity of alveolar membranes, leading to accumulation of mitochondrial ROS and subsequent activation of the NLRP3 inflammatory vesicle, which is consistent with RMPP children with persistent hyperthermia and lung injury (39–41). And the absence of Hex2Cer may affect apoptosis signaling (36). Meanwhile, in our study, the 13 differential metabolites were further validated at follow-up, and all AUCs > 0.80, suggesting that these 13 differential metabolites may be a reliable indicator to differentiate between RMPP and MPP. Significant differences in PCT, NEUT%, LYMPH%, HBDH, LDH, CK, AST, ALT, ALB, ALP, TP, D-Dimer, and FDP were found between patients with RMPP and MPP in the present study (all *P*-values <0.05). Previous studies have shown (42, 43) that PCT, CRP, ESR, ALT, LDH, and D-Dimer levels were higher in the RMPP group, and that these significant markers of inflammation may be predictive of RMPP, further corroborating some of the results in this study. Still in this study there were also significant differences in NEUT%, LYMPH%, HBDH, CK, AST, ALB, ALP, TP, and FDP, so these significantly altered laboratory markers mentioned above could also be used as indicators for early recognition of RMPP.

The extrapulmonary manifestations in children with MPP, such as hepatic impairment, myocardial damage, and abnormal coagulation indices, were also reflected in our correlation analysis, where certain differential lipids (e.g., DG(34:4e), SM(d38:3)) showed associations with relevant laboratory markers. However, these associations should be interpreted with caution. Our findings do not establish a direct mechanistic role of lipids in extrapulmonary pathophysiology. Rather, these lipids may serve as associated biomarkers that reflect systemic inflammatory status or metabolic stress in RMPP, which warrants further validation in future longitudinal and mechanistic studies. Whether the lipidomic changes we observed are pathogen-specific remains an open question. Altered surfactant lipid composition and sphingolipid metabolism have also been reported in other pulmonary infections and inflammatory lung diseases. Therefore, it is possible that some of the lipid alterations we detected represent general inflammatory or host-response patterns rather than being strictly specific to Mycoplasma pneumoniae. However, the consistent identification of differential lipids such as DG(34:4e), PC(36:5), and SM(d38:3), and their strong correlations with clinical severity markers in RMPP, suggest that these molecules may have particular diagnostic value in the context of refractory Mycoplasma infection. Future comparative studies including other infectious pneumonias will be required to clarify the specificity of these findings.

It should be acknowledged that some of the lipid changes identified in our study may reflect increased cellular membrane turnover or flux associated with inflammatory cell infiltration, rather than pathogen-specific effects. For instance, glycerophospholipids and sphingolipids are integral components of cell membranes, and their altered abundance in BALF could partly arise from generic inflammatory processes, such as recruitment and activation of neutrophils or macrophages. Nevertheless, the strong and consistent correlations between specific differential lipids (e.g., DG(34:4e), PC(36:5), SM(d38:3)) and clinical severity markers in RMPP suggest that these lipid perturbations may also carry disease-specific diagnostic value. Future mechanistic studies, including cellular composition profiling and absolute lipid quantification, will be required to disentangle the relative contributions of cellular membrane flux and pathogen-driven metabolic reprogramming. Another limitation is that although patients with confirmed secondary infections were excluded, the possibility of undetected co-infections cannot be fully ruled out. Future multicenter studies incorporating more comprehensive pathogen screening will be needed to further validate the specificity of the findings.

The three lipid classes identified as up-regulated in the RMPP group-DG(34:4e), PC(36:5), and SM(d38:3)-are all major constituents of cellular membranes. DG(34:4e) likely represents an ether-linked diacylglycerol, PC(36:5) a PUFA-based phosphatidylcholine species such as PC(16:1/20:4), and SM(d38:3) a typical sphingomyelin species. These molecules are integral to cell membranes and may be elevated as a consequence of increased inflammatory cell infiltration and membrane turnover during infection and inflammation (31, 32). Therefore, their up-regulation may not be specific to Mycoplasma pneumoniae but instead reflect a generic inflammatory exudate. Nevertheless, their consistent elevation and significant correlations with markers of disease severity (e.g., LDH, CK, D-dimer, FDP) suggest that they retain potential clinical value as biomarkers associated with inflammatory burden in RMPP, even if not strictly pathogen-specific.

STUDY LIMITATIONS: This study has several limitations. First, it was conducted at a single center with a relatively small sample size. In addition, common missing values in LC-MS data may affect interpretation in studies with limited sample sizes. Second, although we attempted to minimize dietary influence by implementing a standardized bronchoscopy procedure (including 6 h of preoperative fasting), the effects of dietary individualization and other host-related factors could not be fully excluded due to the young age and poor tolerance of the children. Third, although patients with confirmed secondary infections were excluded, the possibility of undetected co-infections cannot be completely ruled out. Future multicenter studies incorporating more comprehensive pathogen screening will be needed to further validate the specificity of our findings. Another limitation is related to oxygen therapy. Although all patients received only low-flow oxygen or HFNO and none underwent NIV or mechanical ventilation, oxygen exposure may still influence pulmonary lipid metabolism. Future studies should account for ventilation mode and oxygen exposure as potential confounders of BALF lipid composition. Furthermore, bronchoalveolar lavage fluid cell counts and differentials were not systematically measured in our cohort. Given that lipid alterations in BALF may partly reflect the underlying cellular composition (e.g., neutrophil or macrophage predominance), the absence of this information limits our ability to disentangle host inflammatory cell contributions from pathogen-related effects. Finally, the temporal dynamics of lipidomic alterations and their causal relationship with inflammatory responses remain unclear. High-quality, large-scale, multicenter studies from different regions, ideally integrating longitudinal sampling, BALF cytology, and comprehensive pathogen screening, will be necessary to validate the diagnostic potential of lipid metabolites and to clarify their mechanistic role in refractory Mycoplasma pneumoniae pneumonia.

## 6 Conclusion

Compared to plasma and nasopharyngeal secretions, which are usually used to discover biomarkers, BALF fluid can provide more biochemical information and help to reveal disease pathogenesis because it contains compounds close to the site of stress or injury in the lungs. In this study, we analyzed the lipidomic profile of RMPP and MPP in children and their relationship with clinically relevant indicators. Thirteen differential lipid metabolites in BALF were finally identified, which were significantly correlated with disease severity and showed a clear pattern of segregation especially in RMPP and MPP. Two of these lipid compounds, DG SM (d38:3) and DG (34:4e), were positively correlated with HBDH, LDH, CK, D-Dimer, and FDP. These findings provide insights into the systemic metabolism of RMPP in children and its relationship to disease severity, which may help in understanding and managing MP infections. From a clinical perspective, the identification of differential lipid metabolites in BALF provides potential biomarkers that may assist in the early recognition of refractory Mycoplasma pneumoniae pneumonia (RMPP). Conventional laboratory indicators such as CRP or PCT are not always specific or sensitive, whereas lipidomic markers such as DG(34:4e), PC(36:5), and SM(d38:3) demonstrated strong discriminatory power (AUC > 0.8) and consistent correlations with markers of disease severity. These findings suggest that lipid profiling could help clinicians stratify patients at higher risk for refractory disease, prompting earlier escalation of therapy (e.g., adjunctive corticosteroids, immunomodulators) or closer monitoring to prevent complications. Although BALF sampling requires bronchoscopy and may not be routinely performed, the results highlight lipidomic pathways that could potentially be targeted in less invasive biospecimens (e.g., serum or exhaled breath condensates) in the future. Thus, our study contributes to bridging the gap between molecular insights and clinical management by offering candidate biomarkers and mechanistic clues for individualized treatment strategies in pediatric pneumonia.

## Data Availability

The original contributions presented in the study are included in the article/Supplementary Material, further inquiries can be directed to the corresponding author.
