# Supplementary figures and images for: Lipidomics changes in bronchoalveolar lavage fluid of refractory mycoplasma pneumoniae pneumonia: LC-MS-based analysis of potential biomarkers and pathogenesis
Source: Front Pediatr. 2025 Sep 26;13:1655289. doi: 10.3389/fped.2025.1655289 (PMC12512669; doi:10.3389/fped.2025.1655289)

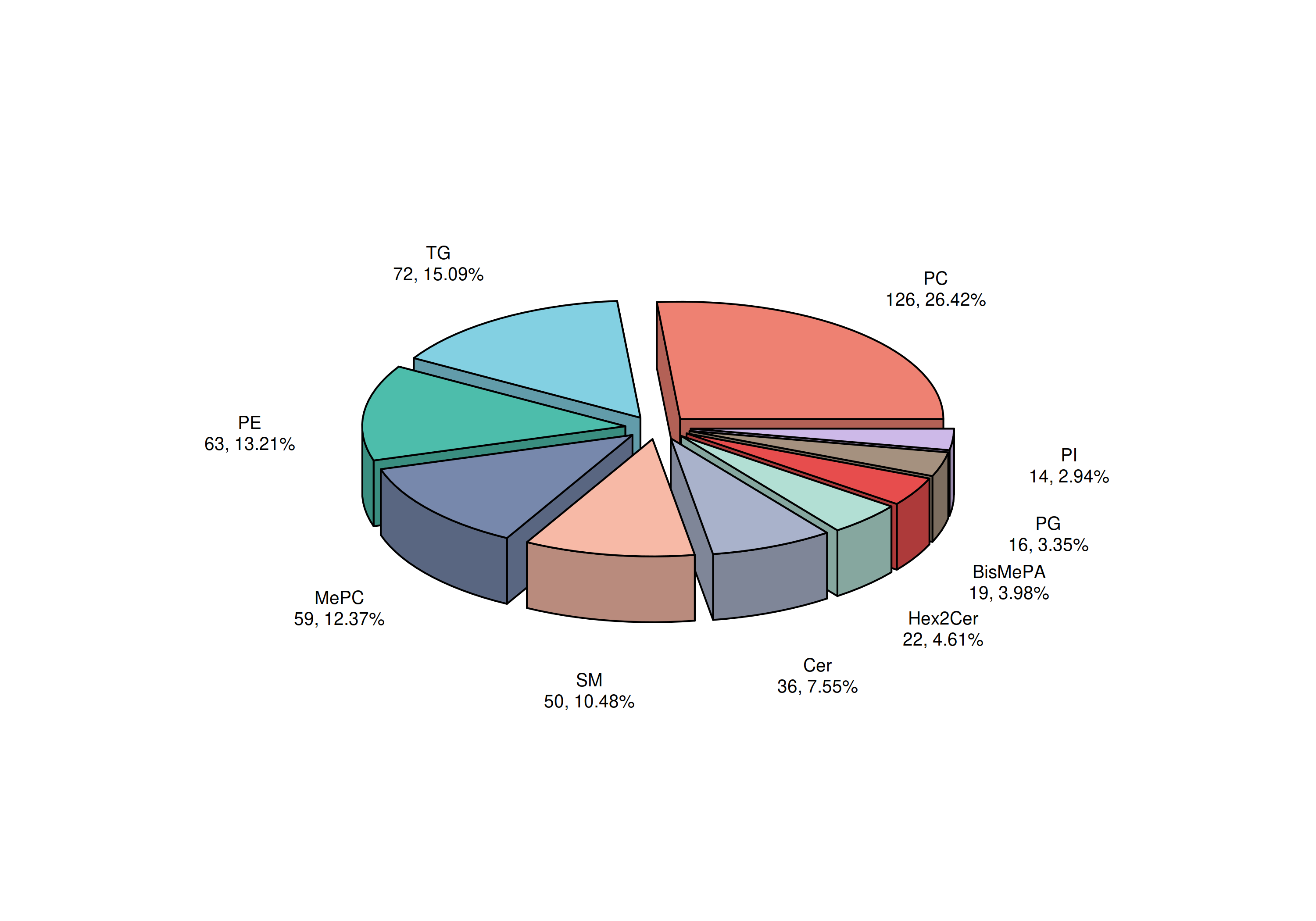

Supplement: Supplementary file 3 [file Image1.tif]
